# The complete mitochondrial genome of the freshwater crab *Tenuilapotamon latilum kaiyangense* Dai et Li,1985 (Decapoda: Brachyura: Potamoidea)

**DOI:** 10.1080/23802359.2021.1955766

**Published:** 2021-07-19

**Authors:** Meng-jun Zhao, Xiao-Juan Zhou, Qian Yang, Xian-Min Zhou, Jie-xin Zou

**Affiliations:** a Research lab of Freshwater Crustacean Decapoda and Paragonimus, School of Basic Medical Sciences, Nanchang University, Nanchang, PR China; b Department of Parasitology, School of Basic Medical Sciences, Nanchang University, Nanchang, PR China; c Key Laboratory of Poyang Lake Environment and Resource Utilization, Ministry of Education, Nanchang University, Nanchang, PR China

**Keywords:** Brachyuran, mitochondrial genome, *Tenuilapotamon latilum kaiyangense*, phylogenetics

## Abstract

We first reported the complete mitochondrial genome of *Tenuilapotamon latilum kaiyangense* (Decapoda: Brachyura: Potamoidea). The genome is 19,294 bp in length, including 13 protein-coding genes, 22 tRNA genes, 2 rRNA genes, and a control region. The whole mitochondrial genome is characterized by the apparent AT bias (74.19%). This research provides DNA data for further researches on population genetics and phylogenetics.

Genus Tenuilapotamon was established in 1984. It has the most subspecies among freshwater crabs, concentrated in and around Guizhou, China. And the genus *Tenuilapotamon* includes *T. joshuiense*, *T. inflexum*, *T. latilum. T. latilum* also includes *T. latilum latilum*, *T. latilum huishuiense*, *T. latilum bijiense*, *T. latilum anshunense*, *T. latilum kaiyangense* and *T. latilum shuichengense* (Dai 1999). However, there is still lack of the molecular data of genus *Tenuilapotamon* in public databases. According to the report, complete mitochondrial genomes contain enough information to reconstruct the phylogenetic relationship (Dabney et al. 2013). In this study, we first report the complete mitochondrial genome of *T. latilum kaiyangense* and try to clarify its phylogeny status.

An adult specimen of *T. latilum kaiyangense* was collected from Wudong Village, Danjiang Town, Leishan County, Kaili City, Qiandongnan Autonomous Prefecture, Guizhou Province, China (latitude 108.1754 and longitude 26.3837) in 2017. The specimen was deposited at the Laboratory Specimen Library of Freshwater Crustacean Decapoda & Paragonimus, School of Basic Medical Sciences, Nanchang University, Nanchang, Jiangxi, PR China & National Parasite Germplasm Resources Specimen Library of China (Jie-xin Zou jxzou@ncu.edu.cn) under the voucher number NCUMCP4020. The sample was stored in 95% ethanol for fixation before sequence analyses. The complete mitochondrial genome was sequenced by first generation sequencing techniques. Genomic DNA extraction, sequencing, gene annotation, and phylogenetic analyses were performed referred to the ways routinely used before (Plazzi et al. 2013). When constructed phylogenetic trees, the sequences were compared according to the G-INS-I method using Mafft ver.7.215 (Katoh and Standley 2013), and were selected the conserved regions using Gblocks 0.91 b (Castresana 2000). Using MrModeltest ver.2.2, the best-fitting model for Bayesian Inference (BI) analysis was selected by the Akaike information criterion (AIC), and the BI tree was established using MrBayes ver.3.2.6 (Ronquist et al. 2012). The MEGA ver.X.0 (Kumar et al. 2018) was used to select the optimal evolutionary model of Maximum Likelihood (ML) analysis and establish the ML tree.

The complete genome of *T. latilum kaiyangense* is 19,294 bp in length (GenBank accession number: MW788029) and contains 13 protein-coding genes (PCGs), 22 tRNA genes, 2 rRNA genes, and 1 control region (CR). The whole mitochondrial genome is characterized by the apparent AT bias (74.19%). Among those genes, 23 genes are encoded by the H chain and 14 genes are encoded by the L chain. The total length of the coding genes is 14,691 bp, and the total length of the non-coding region is 4,603 bp. There are 20 non-coding regions ranging from 1 to 1304 bp in length, and the longest non-coding region is 12S rRNA and tRNA-Ile. There exist seven overlapping regions, the length ranging from 1 to 7 bp in length and the longest one is located between *ND4* and *ND4L*.

The mitochondrial genome of *T. latilum kaiyangense* contains 13 protein-coding genes. Among them, *ND1*, *ND4*, *ND4L* and *ND5* are encoded in the L chain, and the remaining 9 protein-coding genes are encoded in the H chain. The initiation codon of *COX1*, *COX2*, *COX3*, *ATP8*, *ND2*, *ND4*, *ND4L*, *ND5* and *CYTB* is ATG, the initiation codon of *ATP6* and *ND1* is ATA, the initiation codon of *ND3* is ATC and the initiation codon of *ND6* is ATT. The stop codon of all genes is TAA, except *ATP8* is TAG and *COX2*, *CYTB* and *ND5* are incomplete T. The average A + T content of protein coding genes is 71.84%, of which the A + T content of ATP8 is the highest at 81.76%, and the A + T content of *COX1* is the lowest at 64.98%.

The lengths of 22 tRNA genes of *T. latilum kaiyangense* are between 61 bp (tRNA-Arg) and 72 bp (tRNA-Val, tRNA-Met, tRNA-His). All tRNAs genes except for tRNA-Ser (AGN) showed a typical clover structure. The 16S rRNA gene and the 12S rRNA gene are encoded in the L chain, and the lengths are 1321 bp and 826 bp, respectively. The control region of the mitochondrial genome of the *T. latilum kaiyangense* is located between 12S rRNA and tRNA-Ile, with a length of 1304 bp and an AT content of 79.75%.

The phylogenetic position of *T. latilum kaiyangense* in mitogenome relative to other Brachyuran mitogenomes is determined by applying the BI and ML methods on 13 PCGs (Figure 1). We selected the sequence of Kiwa tyleri (GenBank accession numbers：KY423514) from the National Center for Biotechnology Information (NCBI) database as the outgroup. Phylogenetic analysis showed that the two species *T. latilum kaiyangense* and *T.latilum latium* have unique gene sequence, gathered into one branch. The branch is the sister branch of *Sinopotamon*. The complete mitochondrial genome analysis reveals that *Tenuilapotamon* likely to be derived from *Sinopotamon*.

**Figure 1.**
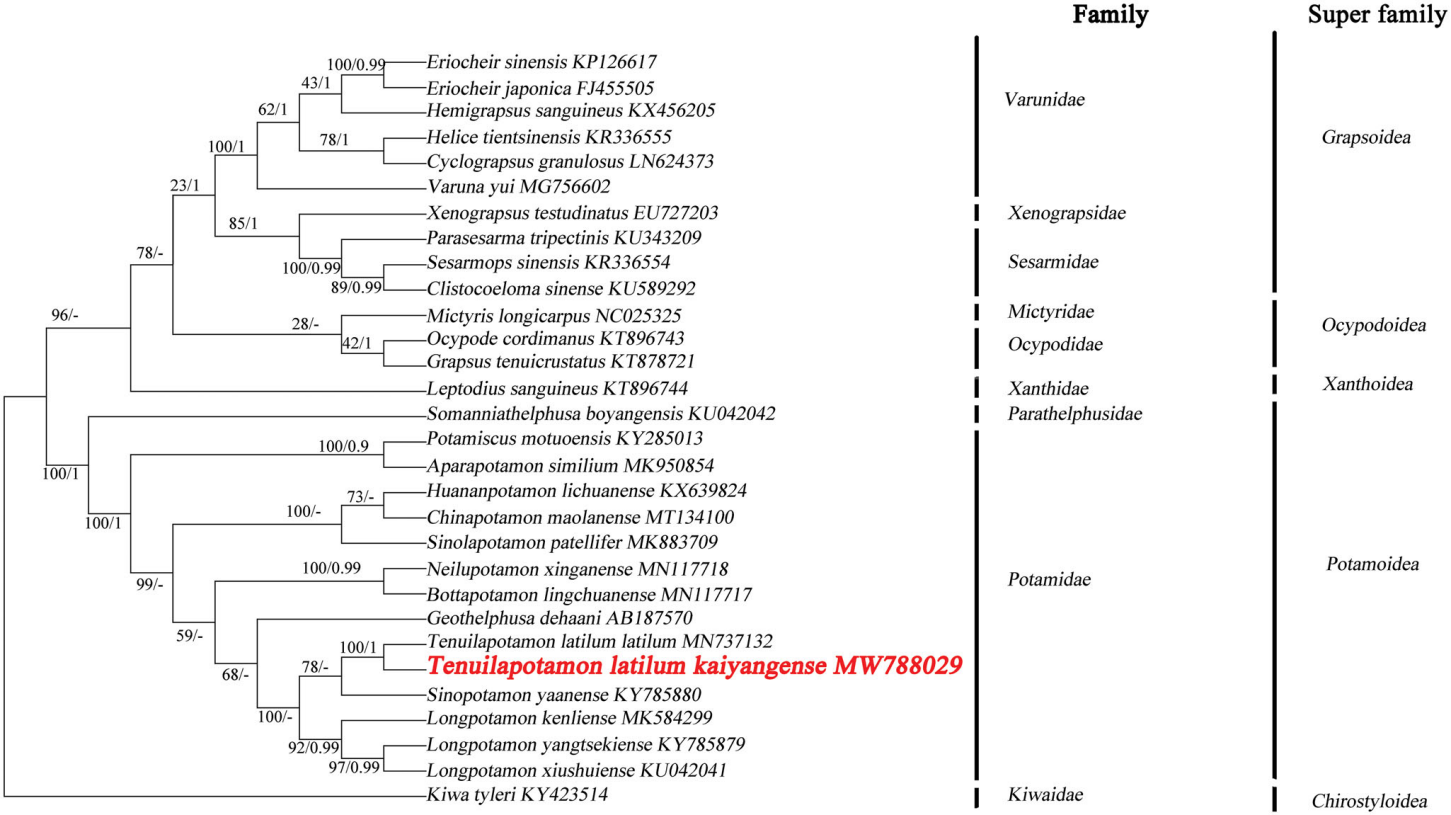
Phylogenetic maximum-likelihood (ML) tree of *Tenuilapotamon latilum kaiyangense* and related brachyurans based on 13 PCGs nucleotide sequences from the mitochondrial genome. The numbers are Bayesian inference (BI) proportions and ML proportions. The differences between the ML and BI trees are indicated by ‘-’.

## Data Availability

The genome sequence data that support the findings of this study are openly available in GenBank of NCBI at (https://www.ncbi.nlm.nih.gov/) under the accession no. MW788029.
